# Prognostic Impact of Renin–Angiotensin System Inhibitors in Revascularized Patients with Acute Myocardial Infarction and Preserved or Mildly Reduced Ejection Fraction: A Retrospective Cohort Study

**DOI:** 10.3390/jcm15072676

**Published:** 2026-04-01

**Authors:** Yanhua Yang, Minqi Liao, Xiaoyu Liu, Zhengwei Jian, Lihua Chen, Yongzhao Yao, Zhiming Yuan, Suxia Guo

**Affiliations:** Department of Cardiology, The Tenth Affiliated Hospital of Southern Medical University (Dongguan People’s Hospital), Southern Medical University, Dongguan 523018, China; yangyanhha@smu.edu.cn (Y.Y.); 13870610249@163.com (M.L.); lxy875834580@163.com (X.L.); okkof@163.com (Z.J.); m13580893736@163.com (L.C.); yyongzhao@outlook.com (Y.Y.); yuanzhimingyzm@163.com (Z.Y.)

**Keywords:** acute myocardial infarction, renin–angiotensin–aldosterone system inhibitors, heart failure, acute coronary syndrome, non-ST-segment elevation myocardial infarction

## Abstract

**Background**: The prognostic value of discharge renin–angiotensin–aldosterone system inhibitor (RASi) therapy in contemporary PCI-treated acute myocardial infarction (AMI) survivors with preserved or mildly reduced left ventricular ejection fraction (LVEF) remains uncertain. **Methods**: A retrospective cohort study of 2530 AMI patients (2019–2022) stratified by RASi use. Exclusion criteria were in-hospital mortality, LVEF < 40%, contraindications to the use of RASis or no percutaneous coronary intervention (PCI). Primary endpoints included heart failure (HF) events, recurrent acute coronary syndrome (ACS), and all-cause mortality. Kaplan–Meier analyses and inverse probability of treatment weighting (IPTW)-weighted Cox models were applied. **Results**: Over a mean follow-up of 49 months, discharge RASi therapy was not associated with all-cause mortality overall, but was associated with fewer HF rehospitalizations (HR 0.62, 95% CI 0.40–0.95; *p* = 0.03). Mortality associations differed by AMI type and hypertension status, particularly for NSTEMI (HR 0.36, 95% CI 0.14–0.91; *p* = 0.03; *p for interaction* = 0.02) and hypertension (HR 0.36, 95% CI 0.15–0.84; *p* = 0.02; *p for interaction* = 0.04). **Conclusions**: In this single-center observational cohort of PCI-treated AMI survivors with LVEF ≥ 40%, discharge RASi therapy was associated with fewer HF rehospitalizations but not with lower overall mortality. Exploratory subgroup analyses suggested potential heterogeneity according to NSTEMI status and hypertension, but these findings should be considered hypothesis-generating and require confirmation.

## 1. Introduction

Cardiovascular diseases (CVDs) are the leading causes of mortality and morbidity worldwide, with ACS often being the initial clinical manifestation of CVD [1]. Following acute myocardial infarction (AMI), the activation of the renin–angiotensin–aldosterone system (RAS) promotes vasoconstriction, sodium retention, sympathetic activation, inflammation, and myocardial remodeling, processes that are mechanistically linked to recurrent ischemic events and progression to heart failure (HF). Accordingly, RAS inhibitors (RASis) have long been a cornerstone of post-MI pharmacotherapy, particularly in patients with left ventricular systolic dysfunction or other high-risk features. Over two decades ago, angiotensin-converting enzyme inhibitor (ACEI) was shown to improve prognosis in postmyocardial infarction patients, particularly those with symptoms of HF, left ventricular ejection fraction (LVEF) < 40%, diabetes, hypertension, and renal insufficiency [2,3]. Early systematic analyses indicated that the use of ACEI was associated with a slight reduction in 30-day mortality, particularly in anterior wall myocardial infarction [4,5,6].

However, the contemporary clinical profile of AMI survivors has changed markedly in the era of rapid percutaneous coronary intervention (PCI), higher rates of complete revascularization, and comprehensive guideline-directed medical therapy. A growing proportion of PCI-treated AMI patients are discharged with preserved or mildly reduced LVEF, potentially lowering absolute event rates and altering the incremental benefit attributable to routine RASi therapy.

The 2025 ACC/AHA/ACEP/NAEMSP/SCAI Guideline for the Management of Patients with Acute Coronary Syndrome provides updated recommendations for RASi therapy: ① a Class I indication for high-risk ACS patients (LVEF ≤ 40%, hypertension, diabetes mellitus, or anterior STEMI) to reduce all-cause mortality and major adverse cardiovascular events (MACEs); and ② a Class IIa indication for non-high-risk ACS patients to reasonably reduce MACEs [7]. Compared with earlier guideline frameworks, the 2025 ACS guideline further stratifies post-AMI RASi recommendations by clinical risk, downgrading use in non-high-risk patients to Class IIa. This shift highlights residual uncertainty, underscoring the need for further evaluation in contemporary PCI-treated survivors with preserved or mildly reduced LVEF. As robust evidence in this specific population remains limited and the magnitude of benefit may vary across clinical strata, additional real-world data are essential to refine hypotheses regarding which subgroups might derive greater benefit from discharge RASi therapy in modern practice.

The objective of this study was to evaluate the association between discharge RASi therapy and long-term clinical outcomes (including HF rehospitalization, recurrent ACS rehospitalization, and all-cause mortality) in a contemporary PCI-treated AMI cohort with LVEF ≥ 40%, using propensity score weighting to mitigate baseline imbalances, and to explore potential heterogeneity of association across predefined clinical subgroups. Given the observational design, findings are interpreted as associational and hypothesis-generating.

## 2. Methods

### 2.1. Study Population and Design

This retrospective cohort study analyzed consecutive patients with AMI, including both STEMI and NSTEMI, admitted to the Tenth Affiliated Hospital of Southern Medical University (Dongguan People’s Hospital) from January 2019 to December 2022.

Inclusion criteria:

- Aged older than 18 years;
- Diagnosed with STEMI or NSTEMI;
- Signed an informed consent form.

Exclusion criteria:

- STEMI patients who did not undergo emergency PCI;
- NSTEMI patients who did not receive PCI according to the GRACE score timeline [1];
- Patients who died during hospitalization;
- Patients with an LVEF < 40%;
- Patients without echocardiography data;
- Patients with contraindications to RASi;
- Patients with advanced malignant tumors.

Exposure status was defined according to the prescription of a RASi (ACEI, angiotensin receptor blocker (ARB), or angiotensin receptor-neprilysin inhibitor (ARNI)) at hospital discharge. Treatment assignment was fixed at discharge and was not reclassified based on medication use during follow-up.

### 2.2. Laboratory and Echocardiographic Examinations

Blood tests were performed in the hospital-certified laboratory according to routine standardized procedures with internal and external quality control. Baseline laboratory parameters, which included complete blood count indices (such as hemoglobin), uric acid, B-type natriuretic peptide (BNP), low-density lipoprotein cholesterol (LDL-c), high-sensitivity troponin I (hs-TnI), and serum creatinine, were extracted from the electronic laboratory information system using the first available measurements obtained during the index hospitalization (typically on admission or within the initial evaluation window).

Transthoracic echocardiography was performed using standard two-dimensional imaging with M-mode measurements (Philips iE33, Philips Medical Systems, Best of the Netherlands) following institutional protocols. LVEF and left ventricular end-diastolic diameter (LVEDD) were obtained from the echocardiographic examination, and LVEF was recorded from the final transthoracic echocardiogram performed prior to hospital discharge. Systolic blood pressure (SBP), diastolic blood pressure (DBP) and heart rate were recorded from the last measurement prior to discharge. Medication data were collected from prescriptions that were continued at discharge and from patients who were followed up after discharge.

### 2.3. Follow-Up and Outcome Assessment

All patients were categorized at discharge into groups based on the use of RASis, which included ACEI, ARB, or ARNI. Treatment assignment was fixed at discharge and was not reclassified based on post-discharge medication changes. Furthermore, dose titration was not analyzed in the present study. The mean follow-up period was 49 months, with a maximum of 74 months.

The primary outcomes were analyzed as separate endpoints, including HF rehospitalization, recurrent ACS rehospitalization, and all-cause mortality. HF events and recurrent ACS were defined as rehospitalizations for HF or acute coronary syndrome. Events were identified through the hospital medical record system and telephone follow-up. All clinical data were sourced from the hospital’s electronic health records, laboratory, and echocardiography systems, and follow-up was conducted via telephone. The data were collected by trained personnel who were blinded to the study’s objectives. Patients lost to follow-up were not included in the data analysis.

### 2.4. Statistical Analysis

Continuous variables are presented as mean ± standard deviation (SD) for normally distributed data and as median with interquartile range (IQR) when not normally distributed. Normality of distribution was assessed using the Shapiro–Wilk test. Categorical variables are expressed as counts and percentages.

Comparisons between groups were performed using Student’s *t*-test for normally distributed continuous variables, the Mann–Whitney U test for non-normally distributed variables, and the χ^2^ test or Fisher’s exact test for categorical variables, as appropriate. Missing data were minimal; therefore, a complete-case analysis was performed.

Because treatment allocation was not randomized and substantial baseline imbalance existed between groups, inverse probability of treatment weighting (IPTW) based on propensity scores was applied as the primary analytic approach to reduce confounding by indication. Propensity scores representing the probability of receiving RASis were estimated using a logistic regression model that included clinically relevant baseline variables associated with treatment allocation: age, sex, hypertension, diabetes, SBP, LVEF, BNP, creatinine, beta-blocker use, etc. Stabilized weights were calculated and applied to generate a weighted cohort.

Covariate balance before and after weighting was evaluated using standardized mean differences (SMD), with an SMD < 0.1 considered indicative of adequate balance. The balance diagnostics are provided in Supplementary Table S1 and Supplementary Figure S1 (Love plot).

Time-to-event outcomes were analyzed using Kaplan–Meier survival curves, and differences between groups were assessed using the log-rank test. The association between discharge RASi therapy and clinical outcomes was evaluated using IPTW-weighted Cox proportional hazards models, with results reported as hazard ratios (HRs) and 95% confidence intervals (CIs).

Prespecified subgroup analyses were performed according to clinical characteristics, including STEMI versus NSTEMI, LVEF categories, diabetes status, hypertension status, and systolic blood pressure levels. Given the limited number of outcome events and the absence of formal correction for multiple comparisons, these subgroup analyses were considered exploratory and hypothesis-generating.

All statistical analyses were performed using R software (version 3.4.3) and EmpowerStats (version 2.17.8). A two-sided *p*-value < 0.05 was considered statistically significant.

## 3. Results

### 3.1. Baseline Characteristics

**Figure 1:**
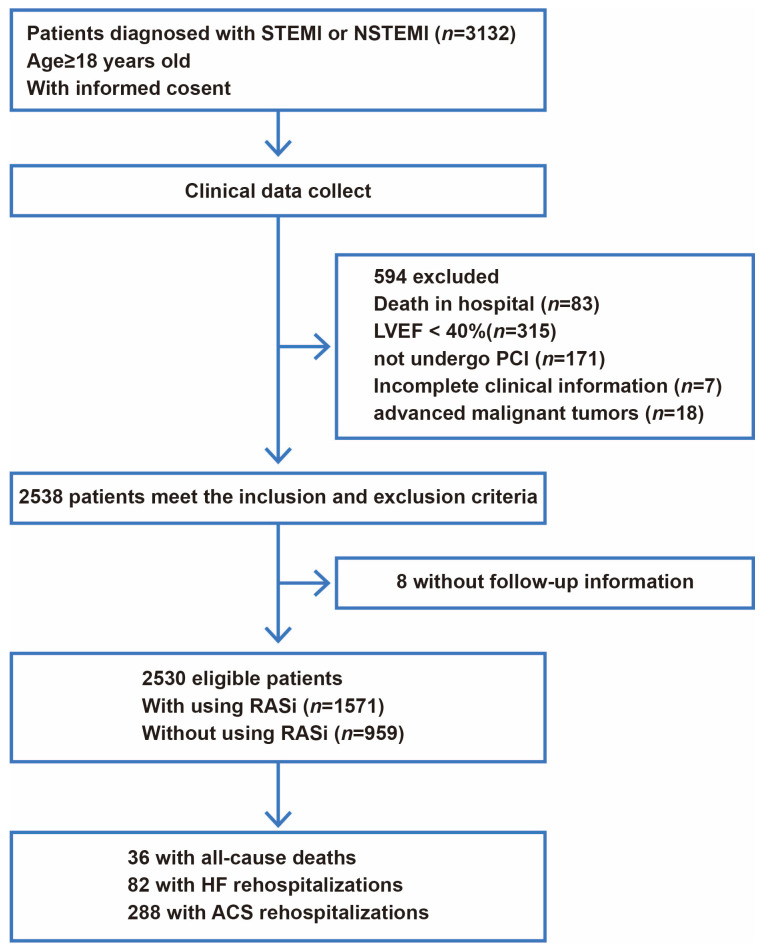
Flow chart of the study.

According to the inclusion criteria of being over 18 years of age and having consecutive cases of STEMI and NSTEMI, 3132 patients were identified. After excluding 594 patients (83 in-hospital deaths, 315 patients with LVEF < 40%, 171 patients who did not undergo PCI at the STEMI and NSTEMI time points or who did not undergo PCI after angiography, 7 patients with incomplete clinical information, and 18 patients with advanced malignant tumors), and 8 patients lost to follow-up, a total of 2530 patients were included in the final analysis. Of these, 1571 patients were treated with RASi, while 959 were not; the study flow chart is presented in Figure 1. The baseline characteristics of patients treated with and without RASi are shown in Table 1. Although several baseline variables showed statistically significant differences between groups, the absolute differences were small and are unlikely to be clinically meaningful. After IPTW, baseline covariates between groups were well balanced (Supplementary Table S1 and Supplementary Figure S1).

In our study cohort, 14 patients (0.595%) had creatinine levels > 265 μmol/L (3 mg/dL). All these patients were dialysis-dependent without hyperkalemia, which did not constitute an absolute contraindication to RASi. Additionally, eight patients (0.32%), all of whom had STEMI, presented with SBP ≤ 90 mmHg on admission; however, their blood pressure normalized by discharge, thus permitting the consideration of RASi therapy.

### 3.2. Overall Outcome Data

The average follow-up period was 49 months, and the longest follow-up period was 74 months. A total of 36 deaths (1.42%), 82 HF events (3.24%), and 288 ACS events (11.38%) occurred during this period, as shown in Table 2.

In the primary IPTW-weighted Cox regression analysis, discharge prescription of RASis was not significantly associated with all-cause mortality in the overall cohort. However, it was associated with a significantly lower risk of HF rehospitalization (HR 0.62, 95% CI 0.40–0.95; *p* = 0.03). Notably, while the IPTW-weighted Cox model indicated a benefit, the corresponding Kaplan–Meier curves did not show a statistically significant difference by the log-rank test (*p* > 0.05).

### 3.3. Subgroup Analysis Results for STEMI and NSTEMI Patients

**Figure 2:**
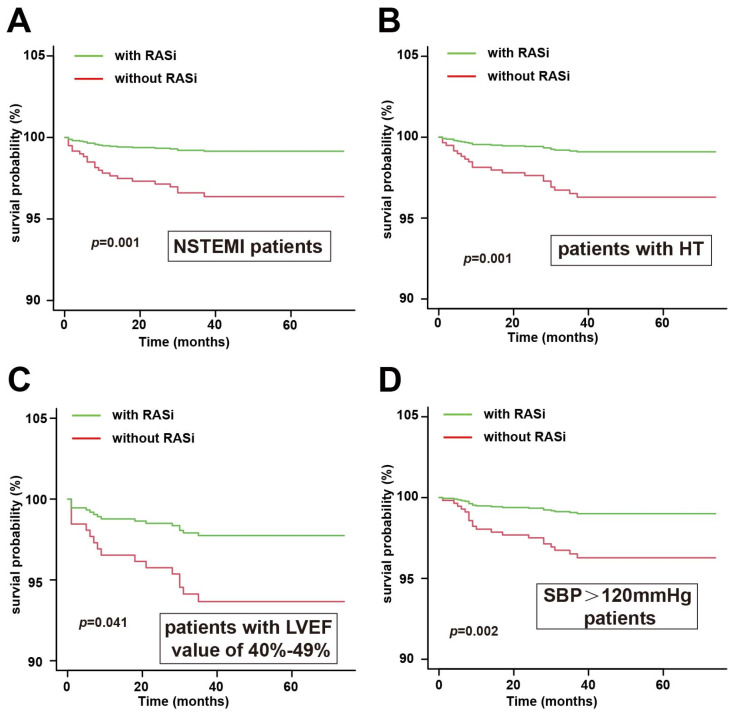
Kaplan–Meier curves for different clinical scenarios. (**A**) All-cause mortality in NSTEMI patients; (**B**) all-cause mortality in patients with hypertension; (**C**) all-cause mortality in patients with LVEF 40–49%; (**D**) all-cause mortality in patients with SBP > 120 mmHg.

Among the 2530 patients, 1382 had STEMI (54.62%), and 1148 had NSTEMI (45.38%). In exploratory subgroup analyses, formal interaction testing indicated significant effect heterogeneity for AMI type status. The interaction between RASi therapy and NSTEMI versus STEMI was statistically significant (*p for interaction* = 0.02). Among NSTEMI patients, discharge RASi therapy was associated with a lower risk of all-cause mortality (HR 0.34, 95% CI 0.13–0.86; *p* = 0.02), as shown in Table 2. The corresponding Kaplan–Meier curves for the use of RASi are shown in Figure 2A.

### 3.4. Stratified Analysis Results for Hypertension

Overall, 1285 patients (50.79%) had hypertension, while 1245 (49.21%) did not. As shown in Table 2, a significant interaction was observed for hypertension status (*p for interaction* = 0.04). Specifically, among patients with hypertension, discharge RASi therapy was associated with lower all-cause mortality (HR 0.32, 95% CI 0.14–0.74; *p* < 0.01). The corresponding survival curves (log-rank *p* value of 0.001) are shown in Figure 2B. Furthermore, in the hypertension subgroup, discharge RASi therapy was also associated with a lower risk of HF rehospitalization (HR 0.55, 95% CI 0.33–0.91; *p* = 0.02). However, the interaction test for HF rehospitalization was not statistically significant (*p for interaction* = 0.53), indicating no clear evidence that this association differed by hypertension status.

### 3.5. Stratified Analysis Results for LVEF 40–49%

This study excluded patients with an LVEF < 40%. There were 406 patients (16.05%) with an LVEF of 40–49%, and 2124 patients (83.95%) with an LVEF ≥ 50%. Subgroup analyses showed that discharge RASi therapy was associated with lower all-cause mortality in patients with mildly reduced LVEF (40–49%) (HR 0.30, 95% CI 0.09–0.96; *p* = 0.04). However, the interaction test was not significant (*p for interaction* = 0.41). The corresponding survival curve (log-rank *p*-value of 0.041) is shown in Figure 2C. Furthermore, there was no significant reduction in HF rehospitalization or ACS rehospitalization. These findings should be interpreted cautiously as exploratory observations rather than confirmatory evidence of differential treatment effects.

### 3.6. SBP Stratification Reveals Divergent Patient Outcomes

Similarly, an association between discharge RASi treatment and lower all-cause mortality was observed among patients with a discharge SBP > 120 mmHg (HR 0.27, 95% CI 0.11–0.67; *p* < 0.01). However, the interaction test across SBP categories was not statistically significant (*p for interaction* = 0.09). The corresponding survival curve for this subgroup (log-rank *p* = 0.041) is shown in Figure 2D. To reduce multiplicity, the primary SBP stratification is presented using the prespecified 120 mmHg cut-off in the main text; additional exploratory thresholds (e.g., 110 and 130 mmHg) are reported in Supplementary Table S2. In contrast, no clinically meaningful or statistically robust pattern was observed for diastolic blood pressure stratification. Therefore, these findings should be interpreted cautiously as exploratory observations rather than confirmatory evidence of differential treatment effects.

**Figure 3:**
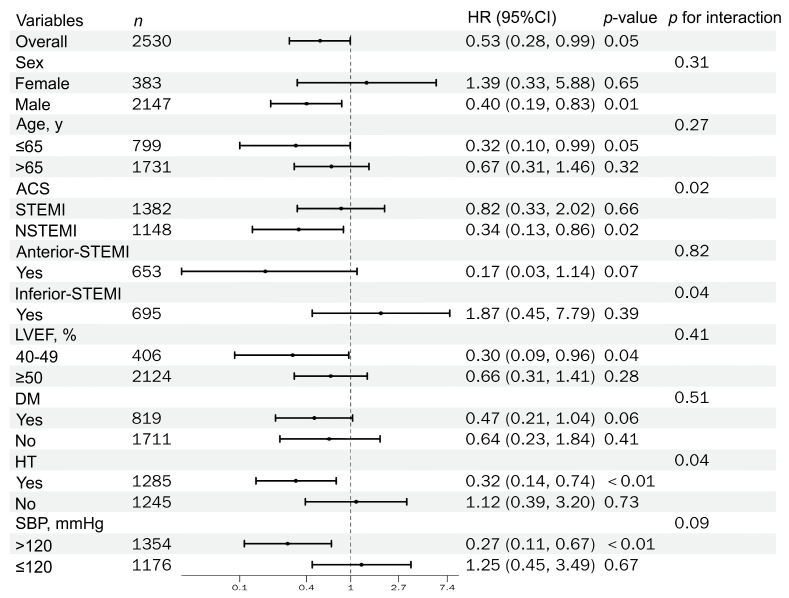
Forest plot of IPTW-weighted HRs for all-cause mortality by prespecified subgroups (with *p for interaction*).

**Figure 4:**
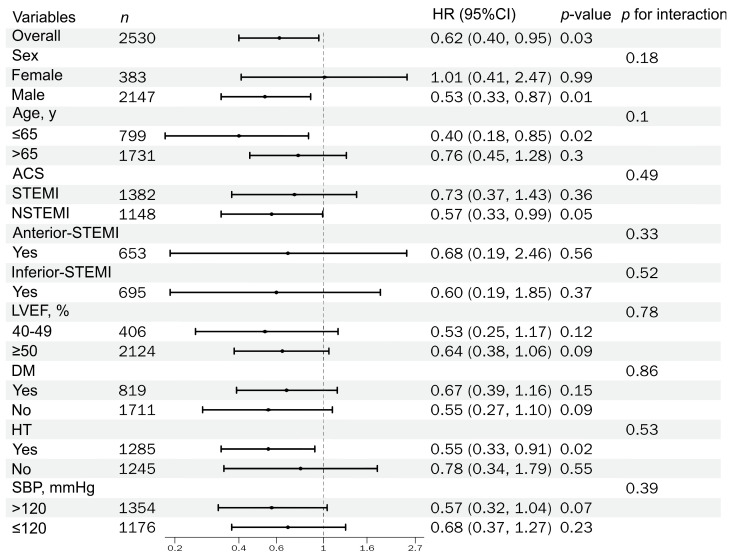
Forest plot of IPTW-weighted HRs for HF rehospitalizations by prespecified subgroups (with *p for interaction*).

**Figure 5:**
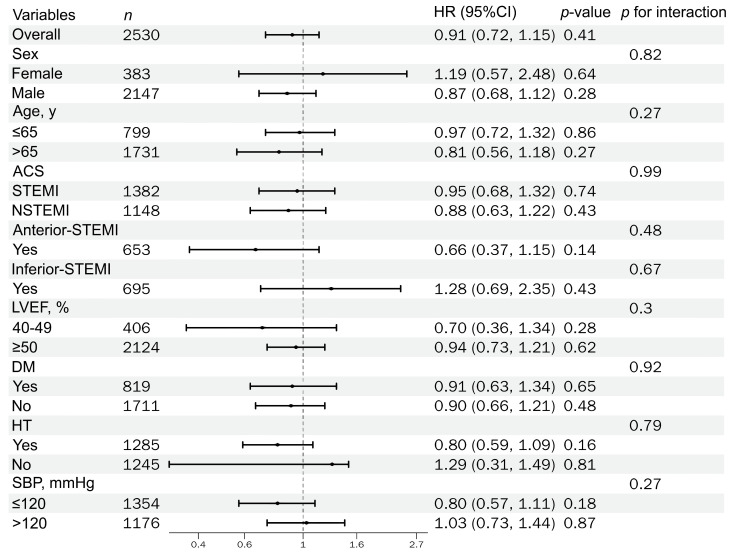
Forest plot of IPTW-weighted HRs for ACS rehospitalizations by prespecified subgroups (with *p for interaction*).

Among AMI patients who underwent PCI within guideline-recommended time windows, had an LVEF ≥ 40%, and survived the index hospitalization, we evaluated the association between discharge RASi therapy and clinical outcomes, including all-cause mortality, HF rehospitalization, and recurrent ACS rehospitalization. Prespecified stratified analyses were conducted across key clinical factors, and the subgroup estimates were derived from IPTW-weighted Cox models; the results are summarized in Figure 3, Figure 4 and Figure 5.

## 4. Discussion

RAS activation after AMI contributes to adverse ventricular remodeling, endothelial dysfunction, and neurohormonal dysregulation, providing a plausible biological rationale for RASi therapy beyond blood pressure lowering. In patients with preserved or mildly reduced LVEF after successful PCI, the absolute risk of remodeling and HF may be lower, and any incremental benefit from routine RASi use may be more dependent on the underlying comorbidity burden and hemodynamic tolerance.

The American Heart Association (AHA) and American College of Cardiology (ACC) issued consistent recommendations [8] based on landmark studies, including the SAVE, AIRE, TRACE, and GISSI-3 trials [5,6,9,10], which shaped treatment strategies post-AMI. However, these trials mainly enrolled thrombolytic-era patients, many with LVEF < 40% and before the routine use of primary PCI. The 2025 ACC/AHA/ACEP/NAEMSP/SCAI Guideline for the Management of Patients with Acute Coronary Syndrome suggests that in patients with ACS who are not considered high risk, an oral RASi is reasonable to reduce MACEs (a downgrade to Class IIa indication) [7]. In the modern era, characterized by timely PCI and a substantial proportion of AMI patients with LVEF > 40%, evaluating the role of RASis in various clinical contexts is increasingly important. This study enrolled 2530 consecutive AMI patients who all underwent PCI (primary PCI for STEMI patients; PCI according to the GRACE score timeline for NSTEMI patients). Among them, 1571 received RASis and 959 did not. Using IPTW-weighted Cox models as the primary analytic approach to mitigate baseline imbalances, we found that discharge RASi therapy was not associated with lower all-cause mortality in the overall cohort. However, RASi use was associated with fewer HF rehospitalizations, while no clear association was observed for recurrent ACS rehospitalization. Notably, interaction testing suggested heterogeneity of the mortality association by AMI type and hypertension status, with a lower mortality risk observed in NSTEMI patients and in those with hypertension. Given the observational design, sparse death events, wide confidence intervals, and multiple subgroup comparisons, these findings should be interpreted as associational and hypothesis-generating.

Evidence in contemporary, lower-risk post-ACS populations has been heterogeneous, with some registries and observational analyses reporting neutral associations of routine RASi use in selected low-risk strata [11]. Large-scale randomized evidence specifically focused on contemporary PCI-treated ACS survivors with preserved or mildly reduced EF remains limited; thus, our results should be warrant confirmation in future prospective studies.

### 4.1. STEMI

As early as 1997, the GUSTO IIB study showed that in centers with advanced interventional capabilities, PCI was superior to rt-PA thrombolysis in improving STEMI outcomes [12]. Subsequent studies confirmed that earlier reperfusion more effectively inhibits ventricular remodeling, reduces HF complications, and improves prognosis [13,14,15].

To enhance early treatment of AMI, the China Chest Pain Center was established in July 2016. By 2023, the standard center’s D-To-W time had shortened to 71 min, and the national average S-To-FMC time was 327.6 min (http://www.chinacpc.org).

In the era of widespread primary PCI, few studies have assessed RASis in STEMI patients. Although the benefits of RASis are well established in patients with left ventricular dysfunction or high-risk features, most recent trials have focused on comparisons between ARNI and ACEI/ARB rather than placebo [16,17,18,19,20]. However, in STEMI patients who receive PCI within 12 h and have preserved or mildly reduced LVEF (>40%), the value of RASis compared to placebo remains uncertain and warrants further investigation.

This study analyzed consecutive AMI patients from Dongguan People’s Hospital (2019–2022). Annual average door-to-wire (D-To-W) times were 71, 63, 56, and 53 min, with a range of 11–192 min; the average symptom-to-first medical contact (S-To-FMC) time was 345.9 min. In this efficient network, timely reperfusion plays a major role in preventing remodeling. Among 1382 STEMI patients, 849 (54.0%) received RASis. As emphasized in the 2025 ACC/AHA Guideline for the Management of Patients with Acute Coronary Syndrome, the initiation of RASis should be guided primarily by high-risk clinical features, such as reduced LVEF, anterior STEMI, hypertension, or diabetes, rather than by whether the presentation is STEMI or not.

In our study of 495 anterior STEMI patients, RASis did not reduce mortality, heart failure rehospitalization, or ACS rehospitalization. The discrepancy may stem from several factors. First, modern care features shorter S-To-FMC and D-to-W times, making timely reperfusion more effective in preventing remodeling than RASis. Second, earlier trials were based on thrombolysis or conservative therapy, which results in greater myocardial damage than primary PCI. Third, most prior patients had reduced LVEF, whereas our cohort had LVEF ≥ 40%, separating the effects of RASis from those primarily benefiting HFrEF patients. Fourth, the sample size of anterior STEMI patients in our study may have been insufficient to detect statistically significant differences.

### 4.2. NSTEMI

Evidence from the international PRAISE registry indicates that after adjustment for clinical and therapeutic variables, patients with STEMI and NSTEMI have largely similar 1-year outcomes, implying that baseline risk and management patterns may matter more than infarct type itself [21].

NSTEMI exhibits significant heterogeneity in symptoms (ranging from shock to mild troponin elevation), coronary lesions (from single-vessel to left main plus triple-vessel disease), and treatment strategies (from simple stenting to use of IABP, ECMO, or LVAD).

The 2023 European AMI guidelines do not offer long-term RASi recommendations specific to NSTEMI [22]. The 2020 guidelines do recommend ACEI/ARB (Class Ia) for patients with LVEF < 40%, diabetes, or chronic kidney disease [1]. However, they lack clear guidance for other scenarios, and the supporting evidence is largely based on older studies [5,23,24].

This study found that in NSTEMI patients, long-term RASi use was associated with reduced all-cause mortality. These findings raise the possibility that NSTEMI patients could derive greater benefit from RASi therapy than STEMI patients, but confirmation in randomized studies is required. First, NSTEMI is often associated with more complex coronary artery disease, such as multivessel or left main involvement, leading to a long-term prognosis that is comparable to or even worse than that of STEMI. Furthermore, NSTEMI patients are often older with multiple comorbidities, making neurohormonal modulation with RASis potentially more valuable. Second, the timing of PCI in NSTEMI is generally guided by risk stratification (e.g., GRACE score), rather than the strict 90 min reperfusion window used in STEMI. This delay in revascularization may allow greater opportunity for RASis to exert mortality-reducing effects through early neurohormonal blockade. Additionally, the reduction in HF rehospitalization observed among NSTEMI patients supports a potential role of RASis in mitigating adverse ventricular remodeling. While these observations highlight potential pathophysiological differences between subgroups, such interpretations remain speculative and require confirmation in future large-scale randomized trials.

### 4.3. AMI with Mildly Reduced LVEF (HFmrEF)

RASis have not been shown to reduce mortality or HF hospitalization in HFpEF patients [25,26]. However, a PARAGON-HF subgroup analysis found ARNI reduced HF hospitalization in those with LVEF < 57% [27], suggesting a potential benefit in patients at the lower end of the LVEF spectrum. Indeed, HFmrEF (LVEF 41% to 49%) accounts for 10% to 20% of all HF cases and broadly resembles HFrEF in its clinical features [28]. Despite this clinical importance, evidence regarding HFmrEF is derived primarily from subgroup analyses rather than dedicated randomized trials. Furthermore, investigations focusing specifically on AMI patients with mildly reduced LVEF remain exceptionally rare.

In our IPTW subgroup analysis, among 406 AMI patients with LVEF 40–49%, discharge RASi therapy was associated with lower all-cause mortality; moreover, interaction testing suggested effect heterogeneity by EF category (*p for interaction* = 0.41). Nevertheless, given the observational design and limited number of deaths, these findings should be interpreted cautiously and considered hypothesis-generating rather than confirmatory. In contrast, patients with preserved LVEF (≥50%) did not appear to demonstrate a similar pattern, suggesting that any potential benefit of RASis in this setting may be concentrated in selected phenotypes, warranting confirmation in future prospective studies.

### 4.4. Impact of Blood Pressure on the Prognosis of AMI Patients

Hypertension further modifies post-AMI risk through chronic vascular injury, endothelial dysfunction, and increased myocardial vulnerability; recent evidence suggests that hypertensive status is associated with more severe cardiovascular injury and biomarker-defined myocardial damage even outside overt acute illness, highlighting the potential relevance of RAS modulation in this subgroup [29]. Consistent with this rationale, our IPTW subgroup analyses suggested potential heterogeneity by hypertension status. Among patients with hypertension, discharge RASi therapy was associated with lower all-cause mortality (HR 0.32, 95% CI 0.14–0.74; *p* < 0.01; *p for interaction* = 0.04).

Zhao Xuedong et al. found that AMI patients with SBP < 100 mmHg undergoing PCI benefited from early RASi use [30]. However, LVEF data were incomplete, and many with SBP < 100 mmHg likely had reduced LVEF, suggesting benefits may mainly apply to those with LVEF < 40%. In our study of patients with LVEF > 40%, RASis reduced mortality only in those with SBP > 120 mmHg. Nevertheless, the interaction test was not statistically significant (*p for interaction* > 0.05), and therefore, there is no definitive evidence of a differential effect between SBP strata, warranting confirmation in future studies. Furthermore, the association observed among patients with higher discharge SBP may reflect greater clinical stability and tolerance of guideline-directed medical therapy.

While antihypertensive therapy is known to prevent major cardiovascular events [31,32], overly aggressive blood pressure reduction may impair myocardial perfusion in AMI. Böhm et al. found both low SBP and DBP worsen AMI prognosis, identifying a DBP threshold of 70 mmHg and an SBP J-curve between 120 and 140 mmHg [33]. Similarly, Vidal-Petiot et al. reported increased mortality risk when SBP < 120 mmHg in stable coronary artery disease patients [34]. These prior observations are consistent with our finding that lower SBP was not associated with a mortality benefit from RASi therapy, possibly due to impaired myocardial perfusion that may attenuate treatment effects. However, our data do not establish a causal mechanism.

Discharge SBP subgroup analyses were prespecified. The primary SBP stratification was <120 vs. ≥120 mmHg, chosen a priori as a clinically familiar cut-off used in contemporary blood pressure classification. Additional SBP thresholds (e.g., 110 and 130 mmHg) were explored and are provided in the Supplementary Materials to reduce multiplicity in the main text.

Contemporary HF management increasingly emphasizes multidrug neurohormonal and hemodynamic modulation across the LVEF spectrum. Recent adjudicated randomized evidence suggests that sodium–glucose cotransporter-2 (SGLT2) inhibitors (such as empagliflozin and dapagliflozin) reduce sudden cardiac death and major cardiovascular outcomes in HF [35], and soluble guanylate cyclase stimulation with vericiguat has been proposed as an option for patients with persistent risk despite guideline-directed therapy, including those with mildly reduced to mildly preserved LVEF [36]. Accordingly, our findings regarding discharge RASi therapy should be interpreted within this evolving therapeutic landscape, and future studies should evaluate the incremental value and optimal sequencing of these agents in contemporary PCI-treated AMI survivors with preserved or mildly reduced LVEF.

This study evaluated a contemporary real-world cohort of PCI-treated AMI survivors with preserved or mildly reduced LVEF (≥40%), a population underrepresented in older landmark trials. The relatively large sample size and long follow-up enabled assessment of clinically meaningful outcomes. In addition, we applied propensity score-based IPTW and reported balance diagnostics to mitigate baseline treatment selection differences, improving the transparency and robustness of the observational comparisons.

However, this study has several limitations. First, this study was observational and treatment allocation was not randomized, which may introduce confounding by indication. Although IPTW based on propensity scores was applied to reduce baseline imbalance, residual confounding cannot be completely excluded. As a single-center study, generalizability to other healthcare systems with different practice patterns, patient characteristics, and follow-up structures may be limited. Second, D-to-W and S-to-FMC intervals were not included, limiting our ability to fully assess the impact of timely reperfusion on outcomes. Third, the complexity and heterogeneity of NSTEMI, including varied coronary lesions and potential incomplete revascularization, could not be fully explored due to the limited sample size; therefore, residual confounding related to coronary anatomy/procedural complexity cannot be excluded. Fourth, the overall number of outcome events was relatively small after applying the study exclusion criteria, which may limit the precision and stability of the effect estimates. A limited number of events may also restrict the number of covariates that can be included in regression models and increase the variability of statistical estimates. Therefore, the observed associations should be interpreted with caution. Fifth, several subgroup analyses were conducted without formal correction for multiple comparisons. Therefore, these subgroup findings should be interpreted as exploratory and hypothesis-generating. Sixth, death is a competing event for rehospitalization; we censored follow-up at death and did not perform formal competing-risk modeling, which may affect estimates for rehospitalization outcomes. Finally, drug-related adverse events and post-discharge dose titration were not captured. Despite these limitations, the study provides a new insight into the use of RASis for AMI patients; prospective multi-center trials are warranted.

## 5. Conclusions

In this single-center retrospective cohort of PCI-treated AMI survivors with LVEF ≥ 40%, discharge RASi therapy was associated with fewer HF rehospitalizations. However, no significant association with all-cause mortality was observed overall in the IPTW analysis. Additionally, exploratory analyses suggested potential heterogeneity in NSTEMI and hypertensive patients. These findings apply to post-discharge PCI-treated survivors with LVEF ≥ 40% and should not be extrapolated to the overall AMI population (e.g., LVEF < 40% or no PCI). Given the observational design and limited death events, the results should be considered hypothesis-generating and warrant confirmation in prospective, multi-center studies.

## Figures and Tables

**Table 1:** Baseline characteristics in AMI patients with and without RASis before IPTW.

	All Patients (*n* = 2530)	Without RASi (*n* = 959)	With RASi (*n* = 1571)	*p* Value
Clinical characteristics and co-morbidities				
Age, y	58.9 ± 13.0	59.6 ± 13.2	58.5 ± 12.9	0.04
Male	2147 (84.9)	801 (83.5)	1346 (85.7)	0.14
STEMI	1382 (54.6)	533 (55.6)	849 (54.0)	0.45
Anterior	495 (19.6)	187 (19.5)	308 (19.6)	0.95
Inferior	537 (21.2)	207 (21.6)	330 (21.0)	0.46
Smoking	1346 (53.2)	501 (52.2)	845 (53.8)	0.45
Alcohol	311 (12.3)	123 (12.8)	188 (12.0)	0.52
Hypertension	1285 (50.8)	370 (38.6)	915 (58.2)	<0.001
Diabetes mellitus	819 (32.4)	326 (34.0)	493 (31.4)	0.17
AF	114 (4.5)	54 (5.6)	60 (3.8)	0.03
Current medication				
ACEI	866 (34.2)	0 (0.0)	866 (55.1)	<0.001
ARB	655 (25.9)	0 (0.0)	655 (41.6)	<0.001
ARNI	52 (2.1)	0 (0.0)	52 (3.3)	<0.001
β-blockers	1826 (72.2)	525 (54.7)	1301 (82.8)	<0.001
Laboratory indices				
Hemoglobin, g/dL	131 (121–142)	131 (118–140)	132 (124–143)	<0.001
Uric acid, μmol/L	356.0 (281.8–447.6)	362.8 (294.0–459.9)	353.2 (277.5–444.1)	<0.001
LDL-C, mmol/L	3.4 (2.9–3.8)	3.4 (2.8–3.8)	3.4 (3.1–3.8)	0.14
hs-TNI, ng/L	59.0 (1.4–153.0)	115.0 (3.2–153.0)	41.6 (1.0–153.0)	0.26
Creatine, μmol/L	75.0 (67.0–85.0)	76.0 (68.0–86.0)	74.9 (66.2–84.0)	<0.001
BNP, ng/L	144.0 (65.0–274.9)	208.0 (87.5–356.0)	120.0 (56.0–229.0)	<0.001
Physical examination				
SBP, mmHg	120 (109–130)	115 (105–125)	122 (112–134)	<0.001
DBP, mmHg	73 (66–79)	70 (64–77)	74 (67–80)	<0.001
Heart rate, bpm	74 (67–78)	74 (68–80)	74 (66–78)	<0.001
Cardiac ultrasound data				
LVEDD, mm	48 (45–51)	57 (44–51)	48 (45–51)	0.007
LVEF, %	61 (55–65)	60 (53–64)	61 (56–66)	<0.001

Continuous variables are presented as mean ± SD when normally distributed and as median with IQR when not normally distributed. Normality of distribution was assessed using the Shapiro–Wilk test. Categorical variables are expressed as counts and percentages.

**Table 2:** HRs for outcomes of patients with AMI using RASis or not after IPTW.

AMI Patient	All-Cause Death			HF Rehospitalizations			ACS Rehospitalizations		
	Events/Total	HR (95% CI)	*p* Value	Events/Total	HR (95% CI)	*p* Value	Events/Total	HR (95% CI)	*p* Value
All	36/2530	0.53 (0.28–0.99)	0.05	82/2530	0.62 (0.40–0.95)	0.03	288/2530	0.91 (0.72–1.15)	0.41
STEMI	15/1382	0.82 (0.33–2.02)	0.66	32/1382	0.73 (0.37–1.43)	0.36	138/1382	0.95 (0.68–1.32)	0.74
NSTEMI	21/1148	0.34 (0.13–0.86)	0.02	50/1148	0.57 (0.33–0.99)	0.05	150/1148	0.88 (0.63–1.22)	0.43
Anterior- STEMI	6/653	0.17 (0.03–1.14)	0.07	14/653	0.68 (0.19–2.46)	0.56	91/653	0.66 (0.37–1.15)	0.14
Inferior- STMEI	8/695	1.87 (0.45–7.79)	0.39	11/695	0.60 (0.19, 1.85)	0.37	35/695	1.28 (0.69–2.35)	0.42
LVEF 40–49%	16/406	0.30 (0.09–0.96)	0.04	31/406	0.53 (0.25, 1.17)	0.12	41/406	0.70 (0.36–1.34)	0.28
LVEF ≥ 50%	20/2124	0.66 (0.31–1.41)	0.28	51/2124	0.64 (0.38, 1.06)	0.09	247/2124	0.94 (0.73, 1.21)	0.62
DM	25/819	0.47 (0.21–1.04)	0.06	52/819	0.67 (0.39–1.16)	0.15	109/819	0.91 (0.63–1.34)	0.65
Non-DM	11/1711	0.64 (0.23–1.84)	0.41	30/1711	0.55 (0.27–1.10)	0.09	179/1711	0.90 (0.66–1.21)	0.48
HT	21/1285	0.32 (0.14, 0.74)	<0.01	58/1285	0.55 (0.33–0.91)	0.02	159/1285	0.80 (0.59–1.09)	0.16
Non-HT	15/1245	1.12 (0.39–3.20)	0.73	24/1245	0.78 (0.34–1.79)	0.55	129/1245	1.29 (0.31–1.49)	0.81
SBP > 120	20/1176	0.27 (0.11–0.67)	<0.01	39/1176	0.57 (0.32–1.04)	0.07	42/1176	1.03 (0.73–1.44)	0.87
SBP ≤ 120	16/1354	1.25 (0.45–3.49)	0.67	43/1354	0.68 (0.37–1.27)	0.23	146/1354	0.80 (0.57–1.11)	0.18

## Data Availability

The data underlying this article will be shared on reasonable request to the corresponding author.

## Supplementary Materials

The following supporting information can be downloaded at: https://www.mdpi.com/article/10.3390/jcm15072676/s1, Figure S1: Love plot: standardized mean differences before and after IPTW; Table S1: Baseline covariates before and after inverse probability of treatment weighting (IPTW); Table S2: Exploratory IPTW-weighted Cox regression results for outcomes using alternative discharge SBP cut-offs (≥110 and ≥130 mmHg).

## Abbreviations

ACEI	Angiotensin-converting enzyme inhibitor
ACS	Acute coronary syndrome
AF	Atrial fibrillation
ARB	Angiotensin receptor blocker
ARNI	Angiotensin receptor neprilysin inhibitor
AMI	Acute myocardial infarction
BNP	B-type natriuretic peptide
Bpm	Beats per minute
CI	Confidence interval
CVD	Cardiovascular disease
DBP	Diastolic blood pressure
DM	Diabetes mellitus
ECMO	Extracorporeal membrane oxygenation
EF	Ejection fraction
GRACE	Global Registry of Acute Coronary Events
HF	Heart failure
HFmrEF	Heart failure with mildly reduced ejection fraction
HFpEF	Heart failure with preserved ejection fraction
HFrEF	Heart failure with reduced ejection fraction
HR	Hazard ratio
HT	Hypertension
IABP	Intra-Aortic Balloon Pump
IPTW	Inverse probability of treatment weighting
LDL-c	Low-density lipoprotein cholesterol
LVAD	Left ventricular assist device
LVEDD	Left ventricular end-diastolic diameter
LVEF	Left ventricular ejection fraction
MACE	Major adverse cardiovascular events
NSTEMI	Non-ST-segment elevation myocardial infarction
PCI	Percutaneous coronary intervention
RASis	Renin–angiotensin–aldosterone system inhibitors
SBP	Systolic blood pressure
STEMI	ST-segment elevation myocardial infarction
hs-TNI	High-sensitivity troponin I
