# Supplementary material for: Prognostic Impact of Renin–Angiotensin System Inhibitors in Revascularized Patients with Acute Myocardial Infarction and Preserved or Mildly Reduced Ejection Fraction: A Retrospective Cohort Study
Source: J Clin Med. 2026 Apr 1;15(7):2676. doi: 10.3390/jcm15072676 (PMC13072846; doi:10.3390/jcm15072676)
Supplement: Supplementary file 1 [file jcm-15-02676-s001.zip › Supplementary Table S1.pdf]

Supplementary Table S1. Baseline covariates before and after inverse probability of treatment weighting (IPTW).

			Pre-IPTW					Post-IPTW				
**Covariate**	**level**	**Overall**	**0**	**1**	***p***	**test**	**SMD**	**0**	**1**	***p***	**test**	**SMD**
n		2530	959	1571				949.30	1590.96			
age (median [IQR])		58.00 [49.00, 68.00]	59.00 [50.00, 69.00]	57.00 [49.00, 68.00]	0.04	nonnorm	0.09	59.00 [50.00, 68.00]	58.00 [50.00, 69.00]	0.75	nonnorm	0.02
male (%)	0	383 (15.1)	158 (16.5)	225 (14.3)	0.16		0.06	140.0 (14.7)	226.4 (14.2)	0.75		0.01
	1	2147 (84.9)	801 (83.5)	1346 (85.7)				809.3 (85.3)	1364.6 (85.8)			
HT (%)	0	1245 (49.2)	589 (61.4)	656 (41.8)	<0.01		0.40	468.0 (49.3)	770.5 (48.4)	0.73		0.02
	1	1285 (50.8)	370 (38.6)	915 (58.2)				481.3 (50.7)	820.5 (51.6)			
DM (%)	0	1711 (67.6)	633 (66.0)	1078 (68.6)	0.19		0.06	637.7 (67.2)	1065.3 (67.0)	0.93		<0.01
	1	819 (32.4)	326 (34.0)	493 (31.4)				311.6 (32.8)	525.7 (33.0)			
AF (%)	0	2416 (95.5)	905 (94.4)	1511 (96.2)	0.04		0.09	905.3 (95.4)	1509.5 (94.9)	0.72		0.02
	1	114 (4.5)	54 (5.6)	60 (3.8)				44.0 (4.6)	81.5 (5.1)			
beta_bloker (%)	0	704 (27.8)	434 (45.3)	270 (17.2)	<0.01		0.64	265.1 (27.9)	440.6 (27.7)	0.92		0.01
	1	1826 (72.2)	525 (54.7)	1301 (82.8)				684.2 (72.1)	1150.4 (72.3)			
Cr (median [IQR])		75.00 [67.00, 85.00]	76.00 [68.00, 86.05]	74.90 [66.25, 84.00]	0.01	nonnorm	0.16	75.00 [68.00, 84.00]	76.00 [67.00, 86.36]	0.42	nonnorm	0.09

Covariate	level	Pre-IPTW						Post-IPTW				
		Overall	0	1	*p*	test	SMD	0	1	*p*	test	SMD
BNP (median [IQR])		144.00 [65.00, 274.92]	208.00 [87.50, 356.00]	120.00 [56.00, 229.00]	<0.01	nonnorm	0.35	166.54 [75.41, 301.02]	143.00 [63.00, 285.00]	0.04	nonnorm	0.08
SBP (median [IQR])		120.00 [109.00, 130.00]	115.00 [105.00, 125.00]	122.00 [112.00, 134.00]	<0.01	nonnorm	0.48	120.00 [109.00, 131.00]	120.00 [110.00, 130.00]	0.64	nonnorm	0.03
LVEF (median [IQR])		61.00 [55.00, 65.00]	60.00 [53.00, 64.00]	61.00 [56.00, 66.00]	<0.01	nonnorm	0.22	60.00 [55.00, 65.00]	60.00 [54.00, 65.00]	0.66	nonnorm	0.01
HB (median [IQR])		131.00 [121.00, 142.00]	131.00 [118.00, 140.00]	132.00 [124.00, 143.00]	<0.01	nonnorm	0.26	131.72 [121.00, 142.00]	131.00 [121.00, 142.00]	0.55	nonnorm	0.02
UA (median [IQR])		356.00 [281.75, 447.60]	362.80 [294.00, 459.85]	353.20 [277.50, 444.15]	0.01	nonnorm	0.16	355.98 [281.20, 441.36]	360.75 [284.45, 448.10]	0.66	nonnorm	0.01
DBP (median [IQR])		73.00 [66.00, 79.00]	70.00 [64.00, 77.00]	74.00 [67.00, 80.00]	<0.01	nonnorm	0.37	73.00 [66.00, 79.00]	73.00 [66.00, 79.00]	0.93	nonnorm	0.02
HR (median [IQR])		74.00 [67.00, 78.00]	74.00 [68.00, 80.00]	74.00 [66.00, 78.00]	<0.01	nonnorm	0.15	74.00 [67.00, 78.00]	74.00 [67.00, 79.00]	1.00	nonnorm	0.03
LVEDD (median [IQR])		48.00 [45.00, 51.00]	47.00 [44.00, 51.00]	48.00 [45.00, 51.00]	0.01	nonnorm	0.11	48.00 [45.00, 51.00]	48.00 [45.00, 51.00]	0.91	nonnorm	0.01

IPTW, inverse probability of treatment weighting
