# Supplementary material for: Prognostic Impact of Renin–Angiotensin System Inhibitors in Revascularized Patients with Acute Myocardial Infarction and Preserved or Mildly Reduced Ejection Fraction: A Retrospective Cohort Study
Source: J Clin Med. 2026 Apr 1;15(7):2676. doi: 10.3390/jcm15072676 (PMC13072846; doi:10.3390/jcm15072676)
Supplement: Supplementary file 1 [file jcm-15-02676-s001.zip › Supplementary Table S2.pdf]

Supplementary Table S2. Exploratory IPTW-weighted Cox regression results for outcomes using alternative discharge SBP cut-offs (≥110 and ≥130 mmHg)

AMI patient	All-Cause Death				HF rehospitalizations				ACS rehospitalizations			
	Events/ Total	HR (95% CI)	*p* Value	*p for interation*	Events/ Total	HR (95% CI)	*p* Value	*p for interation*	Events/ Total	HR (95% CI)	*p* Value	*p for interation*
SBP>110	27/1811	0.41 (0.20-0.84)	0.02	0.22	63/ 1811	0.56 (0.35-0.90)	0.02	0.37	214/18 11	1.33 (0.82-2.17)	0.25	0.04
SBP≤110	9/719	1.29 (0.31-5.38)	0.73		19/ 719	0.89 (0.34-2.33)	0.81		74/719	0.79 (0.60-1.04)	0.25	
SBP>130	14/630	0.30 (0.10-0.87)	0.03	0.24	31/ 630	0.55 (0.28-1.07)	0.08	0.37	78/630	0.97 (0.74-1.29)	0.86	0.27
SBP≤130	22/1900	0.77 (0.34-1.76)	0.54		51/ 1900	0.69 (0.39-1.21)	0.20		210/19 00	0.78 (0.51-1.20)	0.25	
