# Supplementary figures and images for: Prognostic Impact of Renin–Angiotensin System Inhibitors in Revascularized Patients with Acute Myocardial Infarction and Preserved or Mildly Reduced Ejection Fraction: A Retrospective Cohort Study
Source: J Clin Med. 2026 Apr 1;15(7):2676. doi: 10.3390/jcm15072676 (PMC13072846; doi:10.3390/jcm15072676)

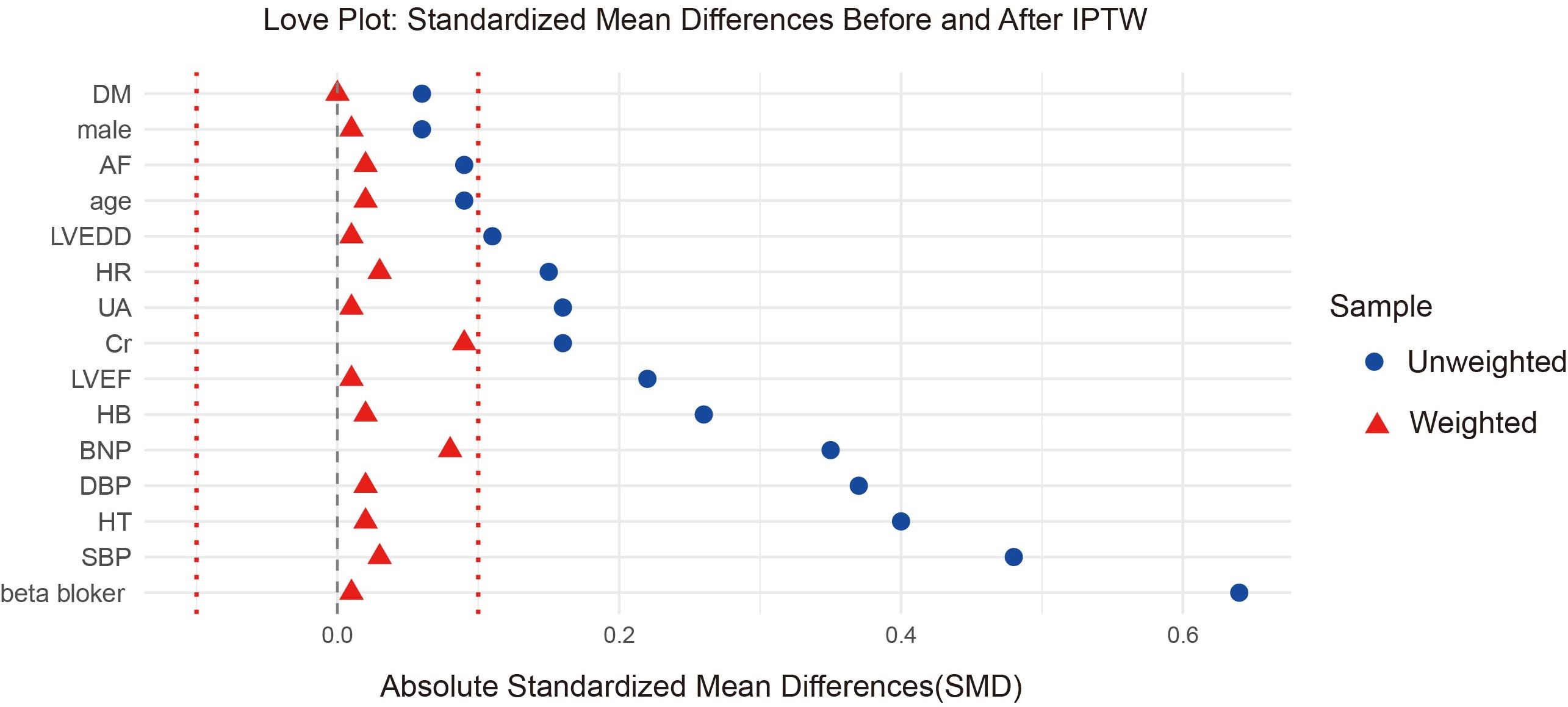

Supplement: Supplementary file 1 [file jcm-15-02676-s001.zip › Figure S1.jpg]
